# Social network characteristics and type 2 diabetes self-management among Black/African American men: A cross-sectional analysis of support quality and communication patterns

**DOI:** 10.1016/j.ypmed.2025.108292

**Published:** 2025-04-24

**Authors:** Tyler Prochnow, Megan S. Patterson, Jeong-Hui Park, Ledric D. Sherman, Matthew Lee Smith

**Affiliations:** a School of Public Health, Texas A&M Health Science Center, 212 Adriance Lab Rd., College Station, TX 77843, USA; b Center for Health Equity and Evaluation Research, Texas A&M University, College Station, TX 77843, USA; c Center for Community Health and Aging, Texas A&M University, College Station, TX 77843, USA

**Keywords:** Social networks, Type 2 diabetes, African American men, Self-management

## Abstract

**Objective:**

Social networks and social support are critically important for Black/African American men managing Type 2 diabetes (T2D). This study aims to examine how social network characteristics are associated with T2D self-management among Black/African American men.

**Methods:**

Cross-sectional survey data were collected from Black/African American men with T2D (*n* = 1225) from February Social to June 2024. Network composition included percentages of spouses, children, parents, siblings, friends, extended family, and healthcare providers. Network interaction measures included communication frequency, support quality, and perceptions of network members’ health behaviors. Self-care activities were measured using the Summary of Diabetes Self-Care Activities scale across diet, physical activity, blood sugar testing, and foot care domains. Multiple regression analyses examined associations between network characteristics and each self-management domain while controlling for demographics.

**Results:**

Diabetes-specific communication frequency was consistently positively associated with all self-care activities (β from 0.09 to 0.18,*p* < .05). Having very supportive network members was positively associated with diet (β = 0.17,*p* < .01) and physical activity (β = 0.20,p < .01), though mean social network support showed negative associations with these behaviors (β = −0.13,*p* = .03; β = −0.14,p = .03). Higher percentages of children were associated with better dietary behaviors (β = 0.06,*p* = .04), while having spouses (β = 0.06,p = .04), friends (β = 0.06,*p* = .03), and siblings (β = 0.06,p = .04) was associated with better foot care.

**Conclusions:**

The quality and content of network interactions appear more important than network size for T2D self-management among Black/African American men. Interventions should focus on fostering quality, disease-specific support rather than expanding social networks. Future programs should leverage existing relationships while considering how different network members influence specific aspects of diabetes management.

Type 2 diabetes (T2D) represents a significant public health lenge, particularly affecting Black/African American men who experience disproportionate burden of the disease (Ahmad et al., 2022). Approximately 13 % of Black/African American adults are diagnosed with T2D compared to 8 % of the general population in the United States (US) (Beckles, 2016). This disparity is especially concerning as poor diabetes management can lead to severe complications including cardiovascular disease, kidney failure, and lower limb amputations (Assari et al., 2020; Magliano et al., 2019). Furthermore, T2D remains the seventh leading cause of death in the US, with approximately 1.4 million Americans diagnosed every year (Association AD, 2022). While thorough and consistent self-management is necessary to prevent these complications, and despite growing research on social contexts of disease management, significant gaps remain in understanding how to effectively leverage social support systems to improve self-management behaviors among Black/African American men (Hawkins, 2019). The intersection of masculine identity, cultural norms, and social support in this population presents unique challenges that require investigation and culturally informed interventions (Bhattacharya, 2024).

Social networks and personal communities play a vital role in chronic disease self-management (Vassilev et al., 2014; Schram et al., 2021). Previous research has demonstrated that health behaviors (e.g., exercising, smoking, and eating) and lifestyle changes can spread through networks (Christakis and Fowler, 2007), and social networks contribute to long-term disease management through actions, emotional activities, and support work undertaken by network members (Vassilev et al., 2014). For individuals with T2D specifically, social support from family members and peers has been shown to improve self-management behaviors (Gatlin et al., 2017; Zupa et al., 2022). Research has indicated that T2D is not considered “a single person’s disease,” but rather affects both the person with diabetes and the people in their social environment, particularly family members (Jones et al., 2008).

Research demonstrates that Black/African American men encounter significant barriers to healthcare utilization and chronic disease management, including masculine identity concerns, cultural norms, and experiences of discrimination in healthcare settings (Seawell et al., 2015; Powell et al., 2016), creating distinct challenges for T2D management. These challenges are further intensified by socioeconomic factors, including limited healthcare access (Gilbert et al., 2016; Fields et al., 2015). The unique characteristics of Black/African American extended family, friendship, and congregational support networks significantly shape health behaviors (Taylor et al., 2013), yet traditional masculine norms often conflict with health-promoting practices, creating additional barriers to effective self-management (Seawell et al., 2015; Griffith et al., 2016). For example, seeking medical assistance or adhering to prescribed dietary and exercise regimens may be perceived as signs of vulnerability, potentially compromising engagement with healthcare services and treatment adherence (Griffith et al., 2016; Griffith et al., 2012). The intersection of these masculine identity concerns with racial and cultural factors creates a complex web of challenges in diabetes self-management that requires careful consideration and focused intervention strategies (Sherman and McKyer, 2015; Sherman and Williams, 2018).

The application of social network analysis (SNA) offers a powerful methodological approach for understanding these complex social dynamics. Unlike traditional measures of social support that focus solely on the presence or absence of support, SNA provides detailed insights into network structure, composition, and the specific qualities of relationships that may influence health behaviors (Valente, 2010; Prochnow and Patterson, 2022). SNA can reveal important structural characteristics such as network density, diversity, and centrality, which may all influence self-management behaviors (Prochnow and Patterson, 2022; Prochnow et al., 2020; McCarty et al., 2019). Understanding these network characteristics is particularly important given that Black/African American men often maintain distinct patterns of extended family, friendship, and community connections that differ from other racial, ethnic, and gender groups (Taylor et al., 2013).

Studies have shown that more diverse networks may provide access to a broader range of resources and support types, while denser networks might facilitate stronger social norms around health behaviors (Vassilev et al., 2014; Perry et al., 2016; Perry and Pescosolido, 2015). However, these relationships have not been thoroughly examined in the context of T2D management among Black/African American men, where cultural factors and masculine identities may shape network formation and utilization in unique ways (Bhattacharya, 2024). Previous research has shown that understanding the structure and function of social networks can inform more effective health interventions (Valente, 2017), yet many current diabetes self-management interventions may not be culturally attuned to the social dynamics and needs present among Black/African American men in their daily lives (Hurt et al., 2015; Crabtree et al., 2015).

This study examines the relationship between social network characteristics and self-care practices among Black/African American men with T2D. By examining both the structural and functional aspects of social networks, we can better understand how different network characteristics influence self-care practices. This detailed analysis of social network qualities can provide insights that go beyond traditional measures of social support, offering new perspectives about how to leverage existing social resources to improve T2D outcomes (Prochnow and Patterson, 2022).

## 1. Methods

### 1.1. Study design

This cross-sectional study collected data via a Qualtrics survey administered between February and June 2024. The internet-based survey aimed to assess social networks and self-care behaviors related to T2D among Black/African American men. The sample was obtained through Cloud Research.

### 1.2. Participants and procedures

The study sample consisted of 1225 Black/African American men living with T2D. Study inclusion criteria were: (1) self-identification as Black/African American; (2) identify as male; (3) age 21 years or older; (4) self-reported T2D medical diagnosis; and (5) reside in the US. Potential participants were directed to an internet-based Qualtrics survey link and provided with an Institutional Review Board-approved information sheet. Participation was voluntary, and respondents could withdraw at any time. Three quality/attention checks were included to ensure data integrity and enhance response validity (Curran, 2016). Respondents had to pass all three validity checks to be included in the final sample. This study was approved by Texas A&M University Institutional Review Board (IRB2023–1311 M).

## 2. Measures

### 2.1. Social networks (independent variables)

A multiple name generator approach was used to elicit participants’ social networks (Barrera, 1980; Marin and Hampton, 2007). This comprehensive method allows for a detailed assessment of participants’ personal support networks (egocentric networks) related to their T2D management. Participants were asked to indicate people who: give them advice, they confide in, provide practical support, and make managing their T2D difficult), resulting in a comprehensive list of social network members. Participants could list the same person across multiple prompts when applicable. For each person nominated in their social network, participants are asked to specify their relationship type (spouse, child, parent, friend, sibling, extended family member, healthcare provider, coworker, roommate, neighbor, or other). Participants also indicate whether each network member has T2D themselves (yes, no, I don’t know). Health behaviors of network members are assessed through two key measures: perceived physical activity frequency and healthy eating habits, both rated on a four-point scale (never, rarely, sometimes, often). Perceived supportiveness specific to diabetes management is evaluated using a four-point scale (not at all supportive, a little supportive, sometimes supportive, very supportive). Contact frequency with each network member was measured using a sixpoint scale ranging from several times daily to never. This was used to measure general communication frequency but also frequency of T2D specific communication. Responses for all network members were aggregated to create multiple network-level variables, including network size, proportion of network members by relationship type (i.e., spouse, child, parent, friend, other family member, health care provider), percentage of network members with T2D, relationship heterogeneity (measure of how many different relationships showed up in their network), mean communication frequency, average level of network support, the frequency of diabetes-specific discussions, and perception of members’ health behaviors (eating healthy and being physically active). Due to the compositional nature of network relationship type data (percentages summing to 100 %), centered log-ratio transformations were performed on network composition variables prior to analysis. This transformation addresses the constraints and dependencies inherent in compositional data while preserving the relative relationship information.

#### 2.1.1. Self-care practices (dependent variables)

The Summary of Diabetes Self-Care Activities (SDSCA) questionnaire was used to assess adherence to self-care practices (Toobert et al., 2000). This 10-item instrument evaluated key domains of diabetes self-management across four primary areas: 1) Healthy Diet (four items): Participants reported their adherence to healthful eating plans and specific dietary recommendations. General diet questions assessed the frequency to which participants followed a healthy eating plan over the past week and month. 2) Physical Activity (two items): Participants indicated their engagement in physical activity, including participation in at least 30 min of continuous activity (including walking) and specific exercise sessions (such as swimming, walking, or biking) separate from daily activities. 3) Blood Glucose Monitoring (two items): Questions assessed the frequency of blood sugar testing. 4) Foot Care (two items): Items evaluated the frequency of foot checks for potential issues. For each item, respondents reported the frequency of self-care activities over the past week (0–7 days). Items were averaged to create a scale score indicating the average number of days per week that respondents engaged in diabetes self-care activities. The SDSCA has demonstrated adequate reliability across diverse cultural contexts, with Cronbach’s alpha values exceeding 0.50 (Gácina et al., 2024).

#### 2.1.2. Demographic variables

Sociodemographic data collected included age, rurality (rural, suburban, urban, or other), educational attainment (less than high school, some college/2-year degree, 4-year degree or higher), employment status (student, employed, unemployed, retired, or unable to work), annual household income (in $25,000 USD increments), marital status (married/partnered, never married, divorced/separated, or widowed), and Body Mass Index (using self-reported height and weight; BMI). These variables were included as controls in subsequent analyses.

## 3. Data analysis

Descriptive statistics, including frequencies, means, and standard deviations, were calculated to summarize the participants’ characteristics. The analysis approach involved a series of regression models examining both overall self-care behaviors and specific components of diabetes management. The first regression model examined overall self-care practices using the composite average SDSCA values as the dependent variable to assess the general influence of network characteristics on diabetes self-management behaviors. Subsequently, additional regression models examined specific associations between network characteristics and diet-related behaviors, physical activity, blood glucose monitoring practices, and foot care practices. All models were adjusted for age, education, urban/rural residential area, employment status, income, marital status, and body mass index. All regression analyses were performed in SPSS v.29.0.0.0 (IBM, 2022).

## 4. Results

### 4.1. Sample characteristics

The study sample consisted of 1225 Black/African American men with an average age of 41.9 years (SD = 14.5) and a mean Body Mass Index of 31.0 (SD = 9.2). Most participants resided in urban areas (52.4 %), followed by suburban (36.1 %) and rural areas (11.1 %), with a small percentage (0.3 %) indicating other residential settings. Most participants were married or partnered (61.1 %), with others reporting never married (27.6 %), divorced or separated (8.8 %), or widowed (2.5 %). Participants reported an average of 2.5 chronic conditions (SD = 1.9). The sample demonstrated economic diversity, with household incomes distributed across multiple income brackets in increments of $25,000. See Table 1 for more information.

Participants reported engaging in self-care activities an average of 4.3 days per week (SD = 1.4). Among specific activities, blood sugar testing was performed most frequently (mean = 4.6, SD = 2.3), followed by diet management (mean = 4.5, SD = 2.0), physical activity (mean = 4.1, SD = 2.1), and foot care (mean = 4.1, SD = 2.4). Analysis of network composition revealed that participants’ social networks contained the highest proportion of friends (18.8 %), followed by healthcare providers (17.7 %), parents (15.1 %), siblings (12.0 %), and spouses (11.7 %). Extended family members (10.6 %) and children (4.9 %) comprised smaller proportions of participants’ networks. On average, 18.6 % of network members also had T2D. Participants’ networks included approximately six individuals (mean = 5.8, SD = 4.3) with moderate relationship heterogeneity (mean = 0.8, SD = 0.4). Regarding network interactions, a majority of network members were perceived as very supportive (64.8 %), with participants reporting high levels of overall social support (mean = 3.6, SD = 0.6). See Table 2 for more information.

### 4.2. Total self-care activities

The model explaining the average frequency of self-care activities explained 16.5 % of the variance (R^2^ = 0.165,*p* < .001). After applying centered log-ratio transformations to address the compositional nature of network data, analysis revealed that higher proportions of children (β = 0.07,*p* = .02) and friends (β = 0.07,p = .02) in one’s network were positively associated with total self-care activities. Other relationship types, including spouses (β = 0.06,*p* = .12), parents (β = −0.03,*p* = .42), siblings (β = 0.05,*p* = .14), extended family (β = 0.03,*p* = .35), and healthcare providers (β = −0.01,*p* = .84), showed no significant associations with total self-care activities after transformation. Network size (β = −0.01,*p* = .63) and relationship heterogeneity (β = 0.002,*p* = .93) also demonstrated no significant relationship with total self-care activities. In network interaction, the proportion of very supportive network members showed the strongest positive association (β = 0.25,*p* < .001), followed by mean frequency of diabetes-specific discussions (β = 0.23,p < .001) and perception of network members eating healthy (healthy eating social norm; β = 0.22,p < .001). Conversely, mean overall social network support (β = −0.21,*p* < .01), mean communication frequency (β = −0.18,p < .01), and proportion of network members contacted less than weekly (β = −0.13,p < .01) demonstrated significant negative associations with total self-care activities. See Table 3 for full model.

### 4.3. Specific self-care activities

#### 4.3.1. Diet self-management

Having a higher percentage of children in one’s network (β = 0.06,*p* = .04) was positively associated with following a healthy eating plan. Less frequent overall communication showed a negative association with dietary adherence (β = −0.15,*p* < .01), while diabetes-specific communication frequency demonstrated a positive relationship (β = 0.13,*p* < .01). Perceptions of network members’ healthy eating habits showed the strongest positive association with dietary management (β = 0.29,*p* < .001). The presence of very supportive network members was positively associated with dietary adherence (β = 0.17,p < .01), though overall mean network support showed a negative relationship (β = −0.13,*p* = .03). See Table 4 for complete results.

#### 4.3.2. Physical activity

No network composition characteristics showed significant associations with physical activity. Regarding interaction patterns, less frequent overall communication showed a negative association with physical activity (β = −0.12,p = .03), while diabetes-specific communication demonstrated a positive relationship (β = 0.09,*p* = .04). Perceptions of network members’ healthy eating habits were positively associated with physical activity (β = 0.11,*p* < .01), though perceptions of network members’ physical activity only approached significance (β = 0.07,*p* = .06). The presence of very supportive network members showed the strongest positive association with physical activity (β = 0.20,*p* < .001), though overall mean network support showed a negative relationship (β = −0.14,*p* = .03). See Table 4 for complete results.

#### 4.3.3. Blood sugar testing

Less frequent overall communication showed a negative association with testing adherence (β = −0.12,*p* = .04), while diabetes-specific communication showed a stronger positive relationship (β = 0.15,*p* < .01). No other network composition, structure, support, or demographic characteristics showed significant associations with blood sugar testing behavior. See Table 4 for complete results.

#### 4.3.4. Foot care

Having spouses (β = 0.06,p = .04), friends (β = 0.06,p = .04), and siblings (β = 0.06,p = .04) in one’s network was positively associated with foot care. Network diversity showed a negative association with foot care behaviors (β = −0.06,*p* = .05). Regarding communication patterns, diabetes-specific discussions showed the strongest positive relationship with foot care (β = 0.18,*p* < .001). Perceptions of network members’ healthy eating habits were also positively associated with foot care (β = 0.12,*p* < .01). Similar to other outcomes, having very supportive network members was beneficial (β = 0.16,p < .01), while higher mean network support showed negative associations (β = −0.18, p < .01). See Table 4 for complete results.

## 5. Discussion

This study provides important insights into how social network characteristics influence diabetes self-management behaviors among Black/African American men with T2D. Findings reveal complex relationships between network composition, perceptions of social norms, interaction patterns, and various self-care activities, with significant implications for both research and intervention development.

Diabetes-specific communication emerged as consistently beneficial across all self-care domains, while other network composition variables showed mixed or negative associations. This aligns with previous research suggesting that focused, disease-specific support may be more effective than general social support for chronic disease management (Vassilev et al., 2014; Zupa et al., 2022). These findings extend prior work by Gatlin (Gatlin et al., 2017) on peer support interventions, suggesting that the content of social interactions may be more critical than the mere presence of support networks. The positive association between diabetes-specific discussions and self-care activities aligns with and extends emerging literature on the critical role of focused health communication within social networks. Perry and Pescosolido (Perry and Pescosolido, 2015) demonstrated that focused health challenges activate specific components of social networks that provide instrumental support for chronic disease management. Schram (Schram et al., 2021) further established that disease-specific communication serves distinct functions beyond general social support, including problemsolving around management challenges and reinforcing health-promoting behaviors. Our results support these findings while specifically demonstrating their applicability to Black/African American men with T2D. This finding takes on additional significance when considered alongside research examining health outcomes among minority populations. Hawkins (Hawkins, 2019) identified that specific health discussions can help bridge cultural gaps in healthcare understanding and facilitate more effective disease management strategies. These findings suggest that interventions aimed at improving diabetes outcomes among Black/African American men should explicitly encourage and facilitate disease-specific discussions within social networks. Such directed communication appears to create opportunities for practical support, knowledge sharing, and accountability that may be particularly valuable for maintaining consistent self-care practices in this population.

Our analysis revealed complex and sometimes counterintuitive relationships between family dynamics and self-care behaviors. Participants who reported a higher percentage of children and friends in their network also reported higher frequencies of self-care activities. The positive association with children in the network has been shown previously for physical activity among Mexican-heritage fathers (Prochnow et al., 2020; Prochnow et al., 2022), and may suggest that fathers are motivated to better manage their diabetes to model healthy behaviors and ensure they remain healthy for their children’s futures. The positive relationship with friends may indicate that peer relationships provide opportunities for mutual encouragement and shared experiences in managing health, creating an environment of normalized health discussions among men. These findings align with conventional wisdom that peer support can foster better diabetes management and self-care behaviors (Jones et al., 2008; Lister et al., 2013). However, spousal and parental presence showed no significant relationship with overall activities, diet, physical activity, or blood sugar testing. These findings may reflect embedded cultural and gender dynamics within Black/African American families, where traditional masculine roles often emphasize strength, independence, and emotional restraint (O’Shan, 2012; High, 2022). Such cultural expectations may create tension between optimal health practices and the desire to maintain one’s social identity and family role (Powell et al., 2016; Griffith et al., 2016; Griffith et al., 2012).

Our analysis uncovered an important distinction between having very supportive network members and overall mean network support levels. While having highly supportive individuals in one’s network was positively associated with self-care activities, higher mean network support across all network members showed negative associations. This distinction may be exceedingly important to note when traditional support measures only provide general feelings of support/aggregate perceptions of overall support instead of network member specific support. By using SNA, results indicated high-quality support from key network members may be more beneficial than broader, but potentially less intensive support distributed across the entire network. This pattern was particularly evident in dietary behaviors and blood sugar testing, extending previous research by Vassilev (Vassilev et al., 2014) on the importance of specific supportive interactions for diabetes management. These findings emphasize the importance of identifying and cultivating relationships with key supporters who can provide consistent, high-quality assistance rather than simply expanding the overall support network.

Our analysis revealed that having healthcare providers in one’s network was not associated with self-care behaviors. This specific finding challenges conventional assumptions about the beneficial role of healthcare providers in disease management and may reflect broader issues in patient-provider relationships. Previous research has documented challenges in healthcare interactions for Black/African American men, including experiences of discrimination, communication barriers, and mistrust of healthcare systems (Powell et al., 2016; Gilbert et al., 2016). Further investigation is needed to understand the complexity of these relationships. The presence of healthcare providers in one’s reported social network may indicate limited access to other sources of social support, suggesting that these individuals may be relying primarily on professional relationships rather than personal connections for support. This potential explanation aligns with research on social isolation and healthcare disparities (McClendon et al., 2021). Future research should explore both the quality of provider relationships and the broader social context in which these relationships exist (Sherman et al., 2023).

Our analysis revealed an important distinction in how social norms within networks influenced different health behaviors. The perception of network members engaging in healthy eating showed significant positive associations with both diet-specific self-care and overall self-management behaviors. However, this pattern did not extend to physical activity, where perceptions of network members’ physical activity habits showed no significant relationship with participants’ physical activity behaviors. This differential impact of social norms may reflect the distinct nature of these health behaviors. While dietary choices are often social activities influenced by shared meals and food-related cultural practices, physical activity presents unique patterns with fewer significant associations with network characteristics. This reduced network influence on physical activity may reflect broader social and environmental factors that extend beyond immediate social network influences (Prochnow and Patterson, 2022; Prochnow et al., 2023). This finding suggests that interventions aimed at increasing physical activity may need to address both social support systems and environmental barriers simultaneously.

### 5.1. Implications

Findings suggest programs aimed at improving diabetes outcomes among Black/African American men should consider how different family and social relationships might be leveraged to support specific aspects of diabetes self-management. The historical and societal context in which many Black/African American men operate often equates discussing health challenges with weakness or vulnerability, creating significant barriers to effective disease management (Powell et al., 2016; Griffith et al., 2016; Griffith et al., 2012). The pressure to embody traditional masculine ideals while managing a chronic condition creates a particularly challenging dynamic within family relationships. Many Black/African American men may perceive seeking or accepting help, even from close family members, as a threat to their role as family providers or leaders (Sutton, 2023). This perception can lead to resistance against well-intentioned family support efforts, potentially explaining the observed negative or null associations in our study (Sutton, 2023). Fields (Fields et al., 2015) noted that these masculine identity concerns often intersect with broader cultural narratives about strength and resilience in Black/African American communities, creating multiple layers of resistance to health-related help-seeking behaviors.

### 5.2. Limitations and future directions

This study has several limitations that should be considered. The cross-sectional design prevents causal inference about the relationships between network characteristics and self-care behaviors. Additionally, self-reported data may be subject to recall bias and social desirability effects, particularly when discussing health behaviors. Future research should employ longitudinal designs to examine how network characteristics and self-care behaviors evolve over time, as suggested by recent studies. Researchers should also consider incorporating objective measures of diabetes management outcomes and exploring how digital health technologies might influence social support networks. Furthermore, the participants in this study were limited to Black/African American men, so it is necessary to examine more evidence with diverse ethnic groups and adequate sample sizes from the same perspective.

## 6. Conclusion

This study makes significant contributions to our understanding of how social networks influence T2D self-management among Black/African American men. The findings suggest that effective programs should focus on fostering quality, disease-specific support rather than simply expanding social networks. As the field moves forward, researchers and practitioners should continue to examine how to effectively promote supportive network interactions while respecting cultural values and gender roles.

## Figures and Tables

**Table 1:** Descriptive characteristics of Black/African American men with type 2 diabetes collected in 2024 (*N* = 1225).

Characteristic	Mean (SD)	N	%
Age (years)	41.9 (±14.5)		
Body mass index (kg/m^2^)	31.0 (±9.2)		
Number of chronic conditions	2.5 (±1.9)		
Residential area			
Urban		642	52.4
Suburban		442	36.1
Rural		136	11.1
Other		4	0.3
Educational attainment			
Some high school, no diploma		20	1.6
High school diploma/GED		263	21.5
Some college, no degree		315	25.8
Technical/vocational training		43	3.5
Associates degree		166	13.6
Bachelor’s degree		311	25.4
Master’s degree		91	7.4
Doctoral degree		14	1.1
Annual household income			
Less than $24,999		140	11.4
$25,000–$49,999		323	26.4
$50,000–$74,999		303	24.7
$75,000–$99,999		223	18.2
$100,000–$124,999		109	8.9
$125,000–$149,999		52	4.2
More than $150,000		74	6.0
Marital status			
Married/partnered		749	61.1
Never married		338	27.6
Divorced/separated		108	8.8
Widowed		31	2.5
Employment status			
Employed		958	78.2
Retired		119	9.7
Not employed		72	5.9
Disabled		54	4.4
Student		23	1.9

Note: SD = Standard Deviation, GED = General Educational Development program, kg/m^2^ = kilograms per meter squared. Total percentages may not equal 100 due to rounding.

**Table 2:** Social network characteristics and self-care activities descriptives among Black/African American men with type 2 diabetes collected in 2024.

Characteristic	Mean	SD
Self-care activities		
Mean self-care activities	4.3	1.4
Healthy diet	4.5	2.0
Physical activity	4.1	2.1
Blood sugar testing	4.6	2.3
Foot care	4.1	2.4
Network composition		
Percent spouse	11.7 %	18.2 %
Percent child	4.9 %	12.7 %
Percent parent	15.1 %	20.0 %
Percent friend	18.8 %	24.1 %
Percent sibling	12.0 %	16.5 %
Percent extended family	10.6 %	16.5 %
Percent healthcare provider	17.7 %	22.4 %
Percent network with T2D	18.6 %	23.0 %
Network structure		
Network size	5.8	4.3
Relationship heterogeneity	0.8	0.4
Network interaction		
Percent talk less than once per week	20.6 %	25.8 %
Mean communication frequency	3.2	1.0
Mean T2D communication frequency	2.4	1.2
Mean perception of physical activity frequency	2.2	0.6
Mean perception of healthy eating frequency	2.3	0.5
Percent very supportive	64.8 %	35.2 %
Mean social network support	3.6	0.6

Note: SD = standard deviation, T2D = type 2 diabetes. Network composition variables represent the percentage of each relationship type in participants’ social networks. Network structure variables include: Network size (total number of people in one’s social network); Relationship heterogeneity (measure of diversity in relationship types within the network, scaled 0–1). Network interaction variables include: Percent talk less than once per week (percentage of network members contacted less than once weekly); Mean communication frequency (average frequency of general communication with network members, scaled 1–6); Mean T2D talk frequency (average frequency of diabetes-specific discussions with network members, scaled 1–6); Mean perception of physical activity frequency (average perception of how often network members engage in physical activity, scaled 1–4 from never to often); Mean perception of healthy eating frequency (average perception of how often network members eat healthily, scaled 1–4 from never to often); Percent very supportive (percentage of network members perceived as “very supportive” of diabetes management); Mean social network support (average level of support across all network members, scaled 1–4 from not at all supportive to very supportive).

**Table 3:** Multiple linear regression results for social network characteristic associations with total self-care activities among Black/African American Men with type 2 diabetes collected in 2024.

Network characteristic	β	*p*-value
Network composition		
Percent spouse	0.05	0.12
Percent child	0.07	0.02
Percent parent	−0.03	0.42
Percent friend	0.07	0.02
Percent sibling	0.05	0.14
Percent extended family	0.03	0.35
Percent healthcare provider	−0.01	0.84
Percent network with T2D	−0.01	0.76
Network structure		
Network size	−0.01	0.63
Relationship heterogeneity	0.002	0.93
Network interaction		
Percent talk less than once per week	−0.13	<0.01
Mean communication frequency	−0.18	<0.01
Mean T2D communication frequency	0.23	<0.001
Mean perception of physical activity frequency	0.07	0.11
Mean perception of healthy eating frequency	0.22	<0.001
Percent very supportive	0.25	<0.001
Mean social network support	−0.21	<0.01

Note: β = standardized regression coefficient, T2D = type 2 diabetes. Adjusted for age, education, urban/rural residential area, employment status, income, marital status, and body mass index. Network composition variables represent the percentage of each relationship type in participants’ social networks. Network structure variables include: Network size (total number of people in one’s social network); Relationship heterogeneity (measure of diversity in relationship types within the network, scaled 0–1). Network interaction variables include: Percent talk less than once per week (percentage of network members contacted less than once weekly); Mean communication frequency (average frequency of general communication with network members, scaled 1–6); Mean T2D talk frequency (average frequency of diabetes-specific discussions with network members, scaled 1–6); Mean perception of physical activity frequency (average perception of how often network members engage in physical activity, scaled 1–4 from never to often); Mean perception of healthy eating frequency (average perception of how often network members eat healthily, scaled 1–4 from never to often); Percent very supportive (percentage of network members perceived as “very supportive” of diabetes management); Mean social network support (average level of support across all network members, scaled 1–4 from not at all supportive to very supportive).

**Table 4:** Multiple Linear Regression Results for Social Network Characteristic Associations with Specific Self-Care Activities Among Black/African American Men with Type 2 Diabetes Collected in 2024.

Variables	Diet		Physical activity		Blood sugar testing		Foot care	
	β	p	β	p	β	p	β	p
Network composition								
Percent spouse	0.02	0.55	0.02	0.54	0.05	0.15	0.06	0.04
Percent child	0.06	0.04	0.02	0.50	0.06	0.06	0.04	0.21
Percent parent	−0.02	0.51	−0.04	0.21	−0.02	0.56	0.02	0.59
Percent friend	0.05	0.12	0.05	0.12	0.06	0.06	0.06	0.04
Percent sibling	0.03	0.41	0.03	0.38	0.03	0.37	0.06	0.04
Percent extended family	0.03	0.29	0.03	0.33	0.01	0.71	0.04	0.21
Percent healthcare provider	−0.02	0.47	−0.01	0.71	−0.06	0.07	0.00	0.98
Percent with T2D	−0.03	0.27	−0.03	0.31	−0.01	0.83	0.03	0.27
Network structure								
Network size	−0.01	0.63	0.04	0.21	0.01	0.83	0.01	0.66
Relationship heterogeneity	0.03	0.35	0.03	0.32	−0.03	0.35	−0.06	0.05
Network interaction								
Percent talk less than once per week	−0.09	0.05	−0.06	0.24	−0.05	0.29	−0.02	0.77
Mean communication frequency	−0.15	0.01	−0.12	0.03	−0.12	0.04	−0.09	0.11
Mean T2D communication frequency	0.13	<0.01	0.09	0.04	0.15	<0.01	0.18	<0.001
Mean perception of physical activity frequency	0.01	0.71	0.07	0.06	0.04	0.29	0.07	0.06
Mean perception of healthy eating frequency	0.29	<0.001	0.11	0.01	0.02	0.60	0.12	<0.01
Percent very supportive	0.17	<0.01	0.20	<0.001	0.09	0.14	0.16	0.01
Mean social network support	−0.13	0.03	−0.14	0.03	0.05	0.44	−0.17	0.01

Note: T2D = type 2 diabetes. This table presents standardized regression coefficients (β) and *p*-values showing the relationship between social network characteristics and diabetes self-management behaviors, controlling for demographic variables (age, education, rurality, employment status, income, marital status, and body mass index). Network composition variables represent the percentage of each relationship type in participants’ social networks. Network structure variables include: Network size (total number of people in one’s social network); Relationship heterogeneity (measure of diversity in relationship types within the network, scaled 0–1). Network interaction variables include: Percent talk less than once per week (percentage of network members contacted less than once weekly); Mean communication frequency (average frequency of general communication with network members, scaled 1–6); Mean T2D talk frequency (average frequency of diabetes-specific discussions with network members, scaled 1–6); Mean perception of physical activity frequency (average perception of how often network members engage in physical activity, scaled 1–4 from never to often); Mean perception of healthy eating frequency (average perception of how often network members eat healthily, scaled 1–4 from never to often); Percent very supportive (percentage of network members perceived as “very supportive” of diabetes management); Mean social network support (average level of support across all network members, scaled 1–4 from not at all supportive to very supportive).

## Data Availability

Data will be made available on request.
